# RNA Nanoparticles Harboring Radioisotopes or Other Imaging Molecules for Spontaneous Tumor Targeting for Early Cancer Diagnosis

**DOI:** 10.59566/isrnn.2025.0201d

**Published:** 2025-08

**Authors:** Daniel W. Binzel, Congcong Xu, Katherine Binzel, Arijit Ghosh, Adam Pippin, Xin Li, Mario Sechi, Michael V. Knopp, Krishan Kumar, Peixuan Guo

**Affiliations:** 1 Center for RNA Nanobiotechnology and Nanomedicine; Division of Pharmaceutics and Pharmacology, College of Pharmacy; James Comprehensive Cancer Center; The Ohio State University, Columbus, Ohio, USA;; 2 Dorothy M. Davis Heart and Lung Research Institute, The Ohio State University, Columbus, Ohio, USA;; 3 Department of Radiology, St. Jude Children’s Research Hopsital, Memphis, Tennessee, USA;; 4 Wright Center of Innovation in Biomedical Imaging and Digital Health; Department of Radiology, College of Medicine, University of Cincinnati, Cincinnati, Ohio, USA;; 5 Department of Medicine, Surgery and Pharmacy, University of Sassari, Via Vienna 2, 07100 Sassari, Italy;; 6 Laboratory for Translational Research in Imaging Pharmaceuticals, The Wright Center of Innovation in Biomedical Imaging, Department of Radiology, The Ohio State University, Columbus, Ohio, USA

**Keywords:** RNA Nanotechnology, PET/CT Imaging, Cancer Diagnosis, Radio-labelling, Prostate Cancer

## Abstract

Cancer patients benefit from significantly higher survival rates if tumors are detected at early stages and prior to metastasis. Positron emission tomography (PET), computed tomography (CT), Single-Photon Emission Computed Tomography (SPECT), and other imaging techniques allow for noninvasive diagnosis of various tumors in relatively short periods. Targeted delivery of radiation is also an important approach in cancer therapy. Due to the dynamic nature of RNA, RNA nanoparticles demonstrate spontaneous tumor targeting, resulting in rapid accumulation in tumors without the use of targeting ligands. Incorporating tumor-targeting ligands on RNA nanoparticles generates enhanced tumor accumulation and targeting. Here a unique technology to specifically label three-way junction (3WJ) RNA nanoparticles is reported to carry radioisotopes or other imaging markers for cancer imaging. Two RNA nanoparticles were constructed to target prostate specific membrane antigen (PSMA) via a PSMA RNA aptamer or conjugated tert-Butyl-DCL (DCL). The spontaneous cancer homing of the RNA complex resulted in the detection of tumors with high sensitivity in mouse models, which can be applied to any cancer subtype at an early stage. Tumor accumulation occurred due to the motile and deformable nature of RNA nanoparticles, allowing for the high tumor accumulation of the imaging reagent via passing rapidly growing and leaky capillaries in the tumor vasculature. Furthermore, RNA nanoparticles conjugated with a NOTA radioisotope chelator were incubated with ^68^Ga in a pH- and temperature-controlled environment to prevent ^68^Ga non-specific interactions with the negatively charged phosphodiester backbone of RNA. The low pH during ^68^Ga^3+^ conjugation neutralized the negative charge of the phosphate backbone on the RNA, ensuring only specific radioisotope chelation to NOTA. To prove the concept of the proposed system, ^68^Ga-labeled 3WJ was tested in a prostate cancer animal model by PET/CT. The ^68^Ga-SF5 3WJ accumulated in and identified prostate cancer tumors with high sensitivity, resolution, specificity, and reliability. The proof-of-concept study reported in this paper is an important step in the direction of developing novel radiotherapeutic agents for various cancers. The radioisotope- or fluorophore-labelled nanoparticles were excreted from the body quickly due to the motile and deformable nature of the RNA complex, thus reducing the chance of toxicity and side effects. This molecular imaging platform, based on RNA nanoparticles, shows great promise in early diagnosis, staging, and precise treatment of any tumor subtype.

## INTRODUCTION

Early tumor detection is a major factor in patient outlook. Furthermore, identification of metastatic sites is crucial for developing treatment plans and understanding the disease state. These remain challenges in oncology and there is a current need for universal tumor identification and advanced imaging techniques that can create a high tumor to background contrast throughout the body. Positron emission tomography/computed tomography (PET/CT) has become a powerful non-invasive medical imaging tool, providing both anatomical and quantitative physiological information in routine clinical and preclinical settings^1,2^. Currently, radionuclides including ^18^Fluorine (t_1/2_ = 109.8 min)^1^, ^11^Carbon (t_1/2_ = 20.3 min), ^68^Gallium (t_1/2_ = 67.7 min)^3,4^, ^13^N (t_1/2_ = 9.96 min), and ^64^Cu (t_1/2_ = 12.7 h)^5^ are widely used in clinical PET imaging. Compared to ^18^F and ^11^C, ^68^Ga^3+^ can be readily obtained from an in-house ^68^Ge/^68^Ga generator at a lower cost, making it particularly attractive for imaging applications^6^. Current research is working to develop radiolabeled tumor ligands to provide a better tumor to background noise ratio for better tumor identification^4,7–9^. However, the dose and circulation time of the small molecule-based radiopharmaceuticals are limited due to systemic toxicity and the lack of sufficient selectivity to tumor cells.

RNA nanotechnology allows for the construction of programmable nanostructures by exploiting intra- and intermolecular RNA interactions, which enables the design of complex structures with well-defined dimensions, shapes, and sizes^10–12^. The advancement of RNA nanotechnology in the past decades demonstrated the uniqueness of RNA in biomedical applications. In particular, the three-way junctions (3WJ) originated from the packaging RNA (pRNA) of phi29 DNA packaging motor has been engineered for the incorporation of various functional modules including imaging probes, therapeutic agents, and targeting ligands^13–17^. The pRNA-3WJ and now the SF5–3WJ have been used to generate a variety of RNA nanoparticles including planar shapes^13,18–20^, 3D prism architectures^21–24^, and branched dendrimeric structures^25,26^. The constructed RNA nanoparticles have been extensively explored for *in vivo* cancer accumulation and therapeutic delivery. Evidence from previous *in vivo* fluorescent biodistribution studies demonstrated cancer targeting capability in myriads of tumor models such as breast cancer, glioblastoma, gastric cancer, prostate cancer, liver cancer, non-small cell lung cancer, and colorectal cancer^15,16,27–33^.

RNA nanoparticles hold promise to serve as probes for more sensitive bioimaging due to their dynamic and motile properties^12,27,28^. As previously shown, RNA nanoparticles have demonstrated rapid and spontaneous tumor targeting within 30 minutes of systemic delivery^34^. The rubbery and dynamic nature of RNA allows for enhanced nanoparticle penetration through leaky tumor vasculature compared to more rigid nanoparticles^27^. As a result over 5% of injected RNA nanoparticle dose accumulated in the tumor microenvironment^35^. Additionally, tumor targeting can be enhanced through the inclusion of RNA aptamers or chemical targeting ligands to target tumor related receptors such as epidermal growth factor receptor (EGFR)^36,37^, epithelial cell adhesion molecule (EpCAM)^38,39^, prostate specific membrane antigen (PSMA)^15^, or asialoglycoprotein receptor^31^. Furthermore, non-tumor accumulated nanoparticle undergoes rapid glomerular filtration in the kidneys to the urine within 1 hour of delivery^27,40^. RNA nanoparticles are safe due to little chance in lasting healthy organ accumulation as their rapid tumor accumulation and body clearance^41^. These properties further provide promise of RNA nanotechnology serving as an imaging agent in tumor diagnosis. Together, RNA nanotechnology provides a high tumor to organ ratio and its adaptability to carry a variety of imaging molecules provides promise to serving as a bio-imaging platform.

In this study, the feasibility of thermostable RNA nanostructures serving as imaging agents in PET/CT and fluorescence imaging is demonstrated. Primarily as a model we develop RNA nanoparticles to target prostate cancer (PCa), specifically PSMA on PCa cells. Two 3WJ RNA nanoparticles were constructed to include either a PSMA RNA aptamer (SF5 3WJ-PSMA) or conjugation of tert-Butyl-DCL (SF5 3WJ-DCL). Tert-Butyl-DCL is a PSMA inhibitor small molecule. By controlling the valency of DCL on 3WJ nanoparticles, we enhanced the specific targeting to PSMA overexpressed PC3 PIP cells. Radiolabeling of SF5 3WJ nanoparticles was accomplished with high chelation efficiency. *In vivo* PET/CT imaging demonstrated the specific targeting of SF5 3WJ-PSMA nanoparticles to tumor. The proof-of-concept study reported in this paper is an important step in the direction of developing novel radiotherapeutic agents for various cancers by conjugating the RNA nanoparticle with a suitable chelating agent and radiolabeling with a radionuclide; the list includes ^177^Lu, ^225^Ac, ^212^Pb, ^213^Bi, ^223^Ra, and ^90^Y. Additional studies demonstrated RNA dendrimer nanoparticles are able to spontaneously target KB tumors in mice through the inclusion of fluorescent markers and using whole-body imaging and *ex vivo* organ imaging.

## RESULTS

### *In vivo* spontaneous tumor targeting by RNA nanoparticles for early cancer diagnosis

The motility and deformability of RNA nanoparticles result in spontaneous tumor accumulation, thus creating advantageous biodistribution profiles^12,27,28,42^. As shown previously in several tumor models, RNA nanoparticles accumulated in tumors with little to no detection in healthy organs due to the rapid renal clearance of non-tumor accumulated RNA nanoparticles^42^. To further test the ability of our RNA nanoparticles, nu/nu mice bearing KB tumors were injected with previously developed RNA dendrimer nanoparticles harboring iFluor 647 labels^25^. Mice were delivered with the RNA dendrimers that allow for inclusion of multiple imaging markers^25^.

**Fig. 1.**
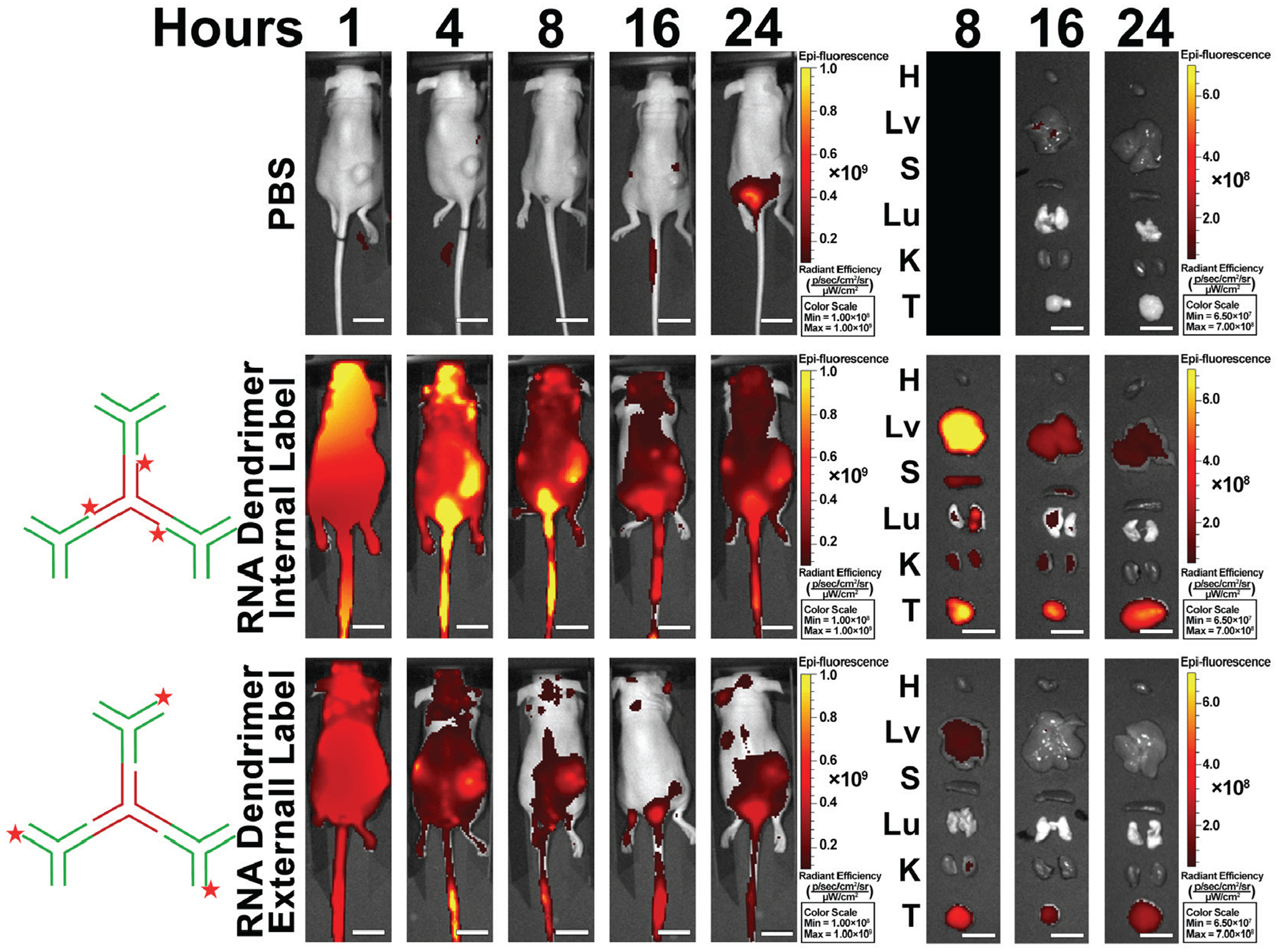
*In vivo* fluorescent time course imaging of animals and organs showing the biodistribution of RNA dendrimers in KB tumor xenograft bearing mice. (H: heart, Lv: liver, S: spleen, Lu: lung, K: kidney, and T: tumor). Scale bar – 1.5 cm.

The G_3_ (third generation) of the dendrimer allowed for the inclusion of three copies of iFluor 647 labels on the interior (G_2_) or exterior (G_3_) of the dendrimer but maintaining the same overall nanoparticle design composed of a pRNA-3WJ at the core with multiple pRNA-3WJs as the G_3_ (Fig. 1 shows designs). Whole body imaging after delivery to nu/nu mice harboring KB tumors demonstrated a rapid tumor accumulation, identifying the tumors at 4 hours (Fig. 1). Interestingly, the location of the near-IR fluorophores of the dendrimer drastically changed the background to tumor signals. *Ex vivo* imaging demonstrated RNA dendrimers quickly cleared from organs in the externally labelled dendrimer and showed tumor only signal at the 16 hour time point (Fig. 1 right panel). These results confirm the spontaneous tumor accumulation of RNA nanoparticles as no ligand was used. Additionally, the results demonstrate the adaptability of the RNA nanoparticle to harbor multiple imaging labels.

### Specific radiolabeling of RNA nanoparticles with high chelation efficacy but no non-specific labelling to the backbone of RNA

We have previously developed 3WJ RNA nanoparticles from Phi29 and SF5 bacteriophage packaging RNAs (pRNA)^13,18^. Both 3WJs display high thermostability and resistance to nuclease degradation by modifying pyramidine nucleotides with 2′-fluorine (2′-F) modifications^40,43^. The 3WJs can be modified to incorporate a variety of therapeutics and tumor targeting ligands.

**Fig. 2.**
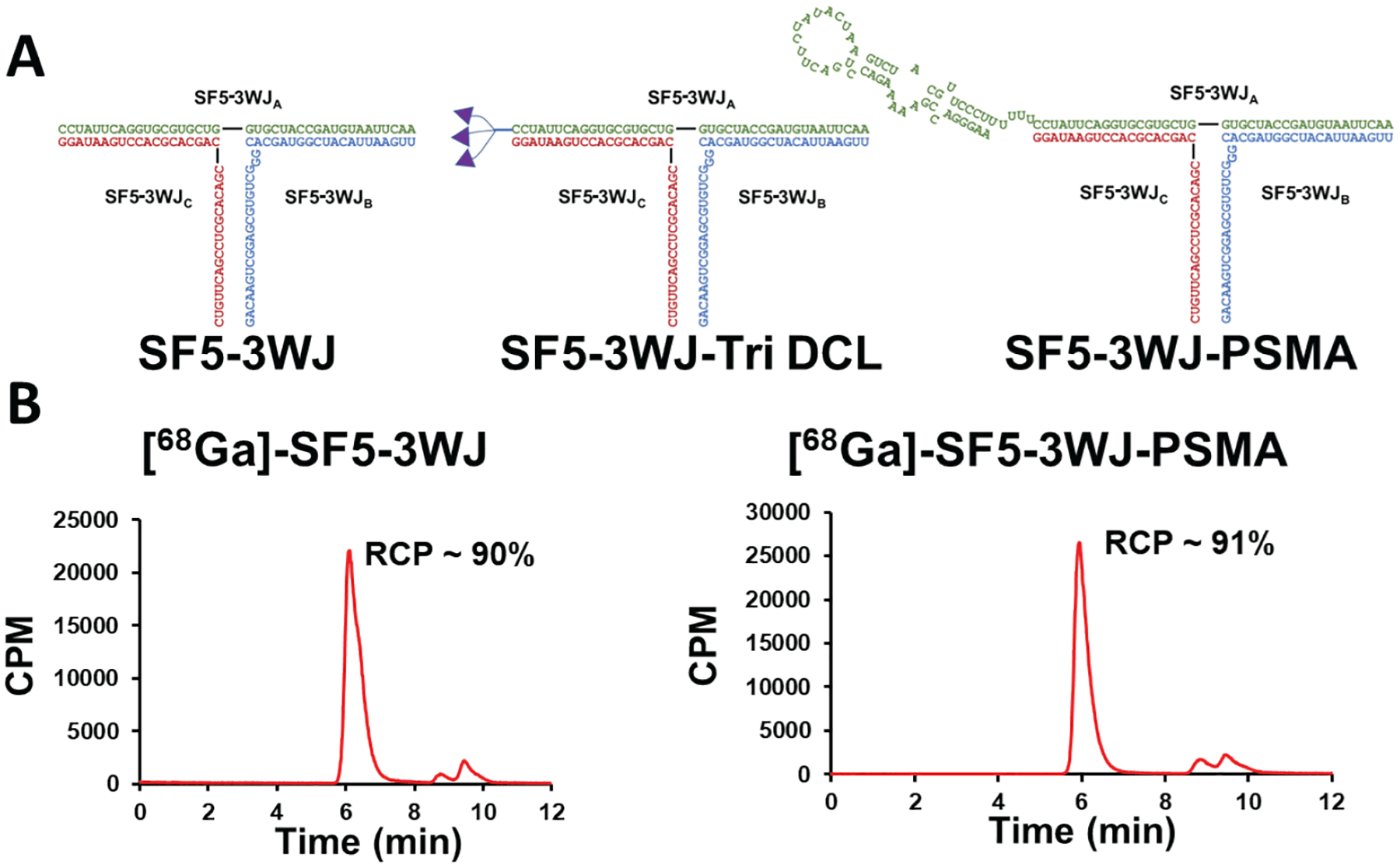
Radiolabeling of RNA nanoparticles as PET imaging agents. **(A)** Design and sequence of SF5 3WJ nanoparticles including three copies of DCL or PSMA RNA aptamer. **(B)** Size exclusion chromatography following [^68^Ga] labelling to SF5 3WJ-NOTA or SF5 3WJ-PSMA-NOTA.

To achieve the PET imaging of cancers using constructed SF5 3WJ nanoparticles (Fig. 2A), the radiolabeling of SF5 3WJ was performed in a “one-pot” self-assembly method. 2, 2′, 2″-(1,4,7-triazacyclononane-1,4,7-triyl)triacetic acid (NOTA) group was conjugated to the SF5 3WJ_C_ strand through NHS ester chemistry to serve as a radioisotope chelator. Following SF5 3WJ assembly, the complex was radiolabeled with ^68^Ga^3+^. To maintain a high chelation efficacy, the reaction was optimized to a pH of 3.6 – 4.5 and temperature of >95°C for 10 – 15 minutes^44,45^. As shown in Fig. 2B, radiolabeled RNA complex was analyzed on size-exclusion chromatography. The retention time of [^68^Ga]-SF5 3WJ-NOTA was 6.44 minutes, and very low amount of [^68^Ga] was detected at 9 min. Similar results were seen for [^68^Ga]-SF5 3WJ-PSMA_apt_ (Fig. 2B). The radiochemical purity (RCP) for SF5 3WJ-NOTA and SF5 3WJ-PSMA-NOTA was determined as 90% and 91%, respectively. The results demonstrated the high chelation efficacy of ^68^Ga on NOTA conjugated RNA nanoparticles.

### Implementation of multivalent DCL on RNA nanoparticles

As PSMA is commonly overexpressed in prostate cancer, it has been used as an ideal target for PCa^46–48^. We have previously developed pRNA 3WJ RNA nanoparticles harboring a truncated PSMA RNA aptamer that results in targeted delivery to PCa cells^15,49^. In this study, we selected a urea-based motif containing lysine (DCL, Fig. 2A) as PSMA-targeting chemical ligand. The DCL contains a primary amine that allows for modification of bio-orthogonal reactive group. To enable the conjugation to RNA, we modified the DCL with azido group *via* NHS ester reaction as shown in Supplemental Fig. 1A. A bifunctional linker NHS-azido was used to mediate the conjugation of DCL on alkyne-modified RNA.

To conjugate the DCL on RNA nanoparticles, we modified RNA with a varying number of alkyne groups at the 5′-end utilizing the doubler and trebler branching phosphoramidites. By creating a branching point, six uracil nucleotides were incorporated as a long linker to impart the flexibility of conjugated ligands (Supplemental Fig. 1). All other pyrimidines (C&U) were 2′-F modified to confer enzymatic stability on RNA. Then the DCL-conjugated strand was used as a component strand for nanoparticle assembly. A new thermodynamically stable 3WJ scaffold re-engineered from SF5 pRNA (SF5 3WJ) was used as shown in Fig. 2A. To increase the thermodynamic stability of nanostructure, the SF5 3WJ core was extended with 11 base pairing (bp) on each helix. Assembly of the SF5 3WJ complex was easily achieved by mixing equimolar ratio of 3WJ_A_, 3WJ_B_, and 3WJ_C_ strands followed by an annealing process. SF5 3WJ_A_ was designed to carry targeting ligand (DCL or PSMA aptamer [PSMA]) while 3WJ_B_ and 3WJ_C_ were conjugated with fluorescent probe or radioisotope chelator, respectively.

### Characterization of RNA nanoparticles with PSMA-targeting ligands

**Fig. 3.**
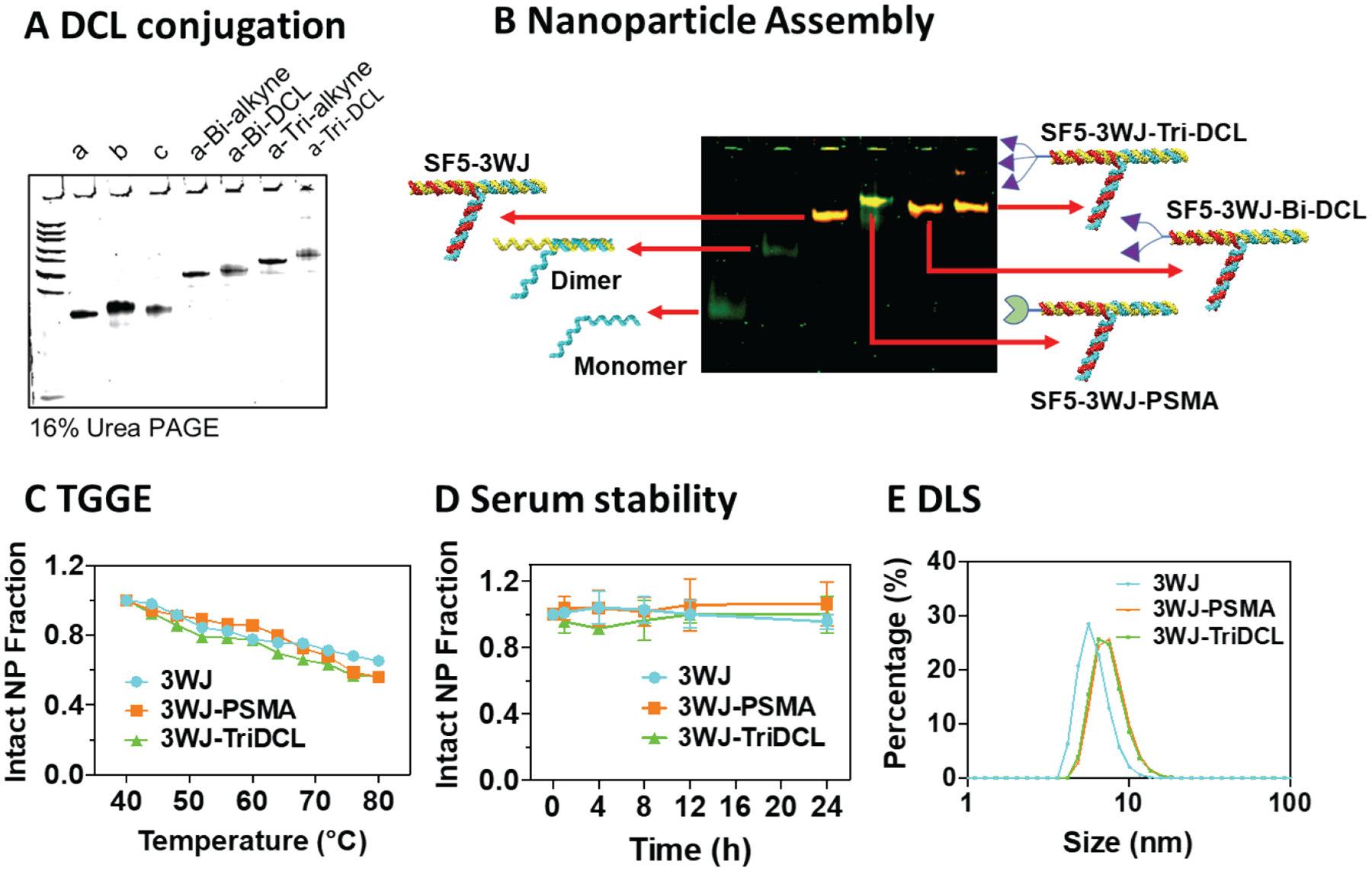
Construction and characterization of PSMA-targeting RNA nanoparticles. **(A)** 16% urea PAGE analysis of synthesized RNA and RNA-DCL conjugates. **(B)** Native PAGE analysis of the step-wise assembly of PSMA-targeting SF5 3WJ nanoparticles. **(C)** TGGE characterization **(D)** Serum stability (n = 3, error bars are presented as mean ± SD) **(E)** Hydrodynamic size of SF5 3WJ-based NPs.

As three individual RNA oligos were incorporated into one nanoparticle complex, a stepwise assembly characterization by 12% native polyacrylamide gel electrophoresis (PAGE) was performed. As shown in Fig. 3B, gel mobility shift was observed from monomer, dimer to 3WJ trimer complex. Assemblies of SF5 3WJ nanoparticles containing bivalent DCL (SF5 3WJ-Bi DCL), trivalent (SF5 3WJ-Tri DCL) and PSMA aptamer (SF5 3WJ-PSMA) were observed with slower gel migration, indicating the successful formation of SF5 3WJ-based complex. As the physicochemical properties of RNA nanoparticles play critical roles in governing the *in vivo* behavior, further characterizations on these RNA nanoparticles regarding thermodynamic stability, enzymatic stability, and size was conducted.

The thermodynamic stability of the PSMA targeting 3WJ nanoparticles was assayed using temperature-gradient gel electrophoresis (TGGE)^40,43^. Temperature gradient was applied perpendicular to the electrical current to determine the melting temperature of the SF5 3WJ complex (Fig. 3C and Supplemental Fig. 2A). With an increasing temperature, more than 50% of each of the SF5 3WJ complexes remained intact, indicating the melting temperature (*T*_*m*_) above the assayed 80°C. This suggests the conjugation of DCL ligands or PSMA aptamer did not hinder the stability of the SF5 3WJ core.

Resistance to enzymatic degradation is another key factor to consider when using RNA as delivery platform. The chemical stability of RNA nanoparticles were assayed through incubation in 10% fetal bovine serum (FBS). Samples were then run on polyacrylamide gels and the intact nanoparticle was quantified. Results showed that each of the SF5 3WJ RNA nanoparticles remained stable at least to 24 h incubation with 10% FBS at 37°C (Fig. 3D and Supplemental Fig. 2B).

Previous studies suggested the importance of the size of nanoparticles in determining the *in vivo* behavior^20^. Dynamic light scattering (DLS) assays were completed on the constructed SF5 3WJ, SF5 3WJ-Tri DCL and SF5 3WJ-PSMA nanoparticles. Slight increase in mean diameter was observed from SF5 3WJ (7.6 ± 1.7 nm) to SF5 3WJ-Tri DCL (9.5 ± 1.2 nm) and SF5 3WJ-PSMA (9.8 ± 1.4 nm) as a result of ligand conjugation (Fig. 3E and Supplemental Fig. 3). The hydrodynamic size of RNA nanoparticles at around 10 nm have been demonstrated to be suitable for *in vivo* tumor targeting with quick tumor accumulation and rapid renal excretion.

### *In vitro* cellular targeting and internalization

**Fig. 4.**
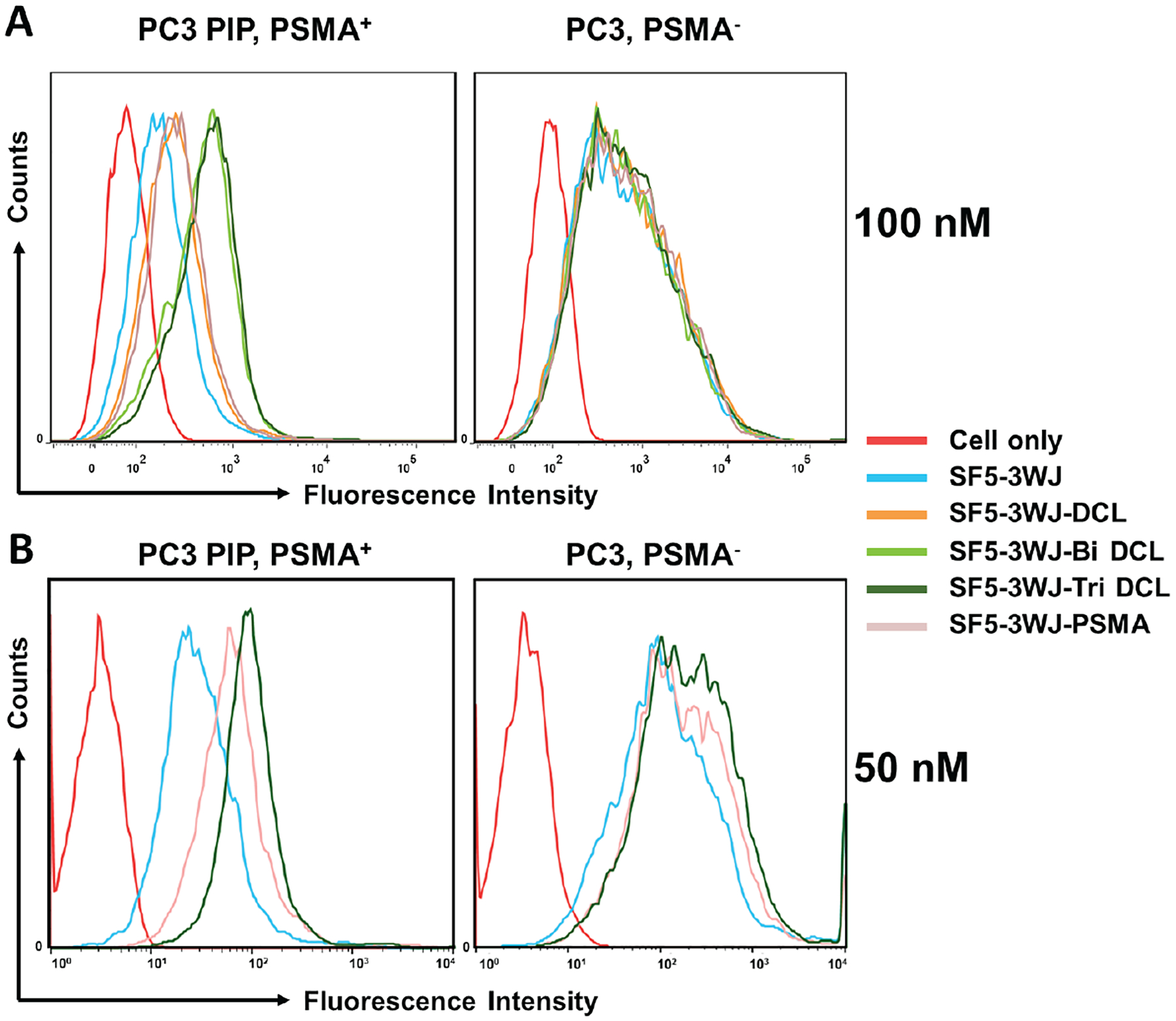
Flow cytometry analysis of RNA nanoparticles (50 nM and 100 nM) binding to PC3 and PC3 PIP cells.

To examine the ability of conjugated DCL to SF5 3WJ nanoparticles, PSMA overexpressing PC3 PIP cells were incubated with AFdye 647 labeled nanoparticles and evaluated by flow cytometry for nanoparticle uptake. Intriguingly, the binding affinity of SF5 3WJ-DCL and SF5 3WJ-PSMA showed similar enhancement in binding compared to 3WJ nanoparticles, while both SF5 3WJ-Bi-DCL and SF5 3WJ-Tri DCL had the strongest binding to PC3 PIP cells (Fig. 4A). In the PSMA negative PC3 control cell line, no significant difference was observed among all groups, suggesting the DCL- and PSMA aptamer-conjugated RNA nanoparticles instill specific targeting and binding to only PSMA on PCa cells. Particularly, the bivalent and trivalent DCL imparted the strongest enhancement in specific binding. To further compare the trivalent DCL with PSMA aptamer, cellular binding assay was completed at a lower 50 nM concentration (Fig. 4B). At both 50 nM and 100 nM concentration, the SF5 3WJ-Tri DCL showed strongest binding to PSMA^+^ PC3 PIP cells, demonstrating the feasibility of using trivalent DCL for enhanced targeting to prostate cancer.

### Internalization of 3WJ nanoparticles to PSMA^+^ and PSMA^−^ PC3 cells

**Fig. 5.**
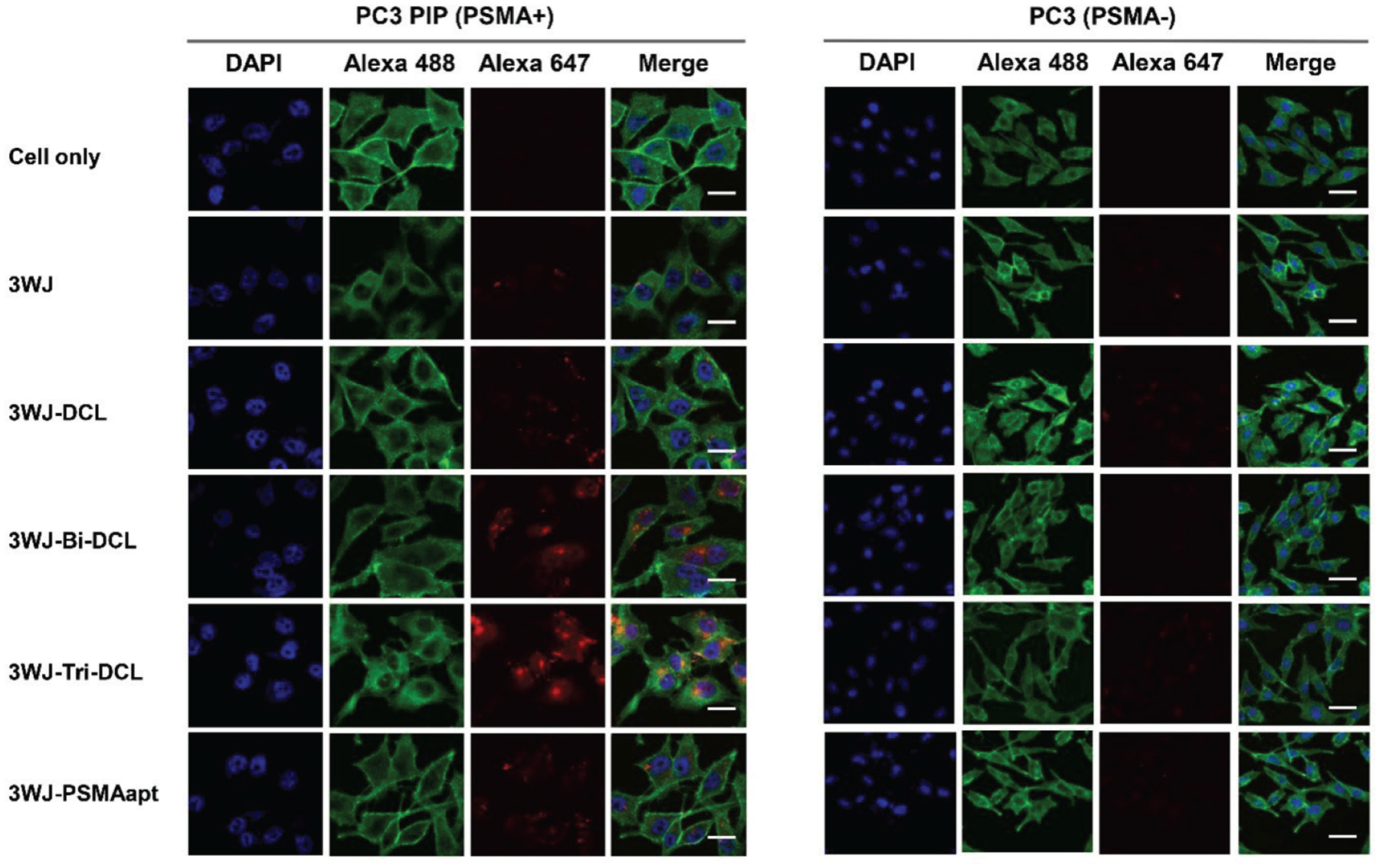
Confocal microscopy imaging of cellular uptake of RNA nanoparticles to PC3 and PC3 PIP cells (blue: nuclei; green: cytoskeleton; red: RNA nanoparticles). Scale bar: left panel – 10 μm, right panel – 20 μm.

To evaluate whether the PSMA-targeting ligands promote the internalization of RNA nanoparticles into PSMA^+^ PCa cells, confocal microscopy imaging was used to analyze fluorescent signals. As shown in Fig. 5, SF5 3WJ-Bi DCL and SF5 3WJ-Tri DCL exhibited substantial cell internalization. In contrast, SF5 3WJ-DCL and SF5 3WJ-PSMA nanoparticles showed less cellular uptake. Confocal images scanned under three separate emission channels clearly showed the difference in cellular uptake among all RNA nanoparticle groups. As the results suggested a strong correlation with DCL valency, it is arguably evidenced that an increased valency of DCL facilitated the receptor-mediated cellular uptake *in vitro*.

### *In vivo* specific tumor targeting by RNA nanoparticles for cancer diagnosis

**Fig. 6.**
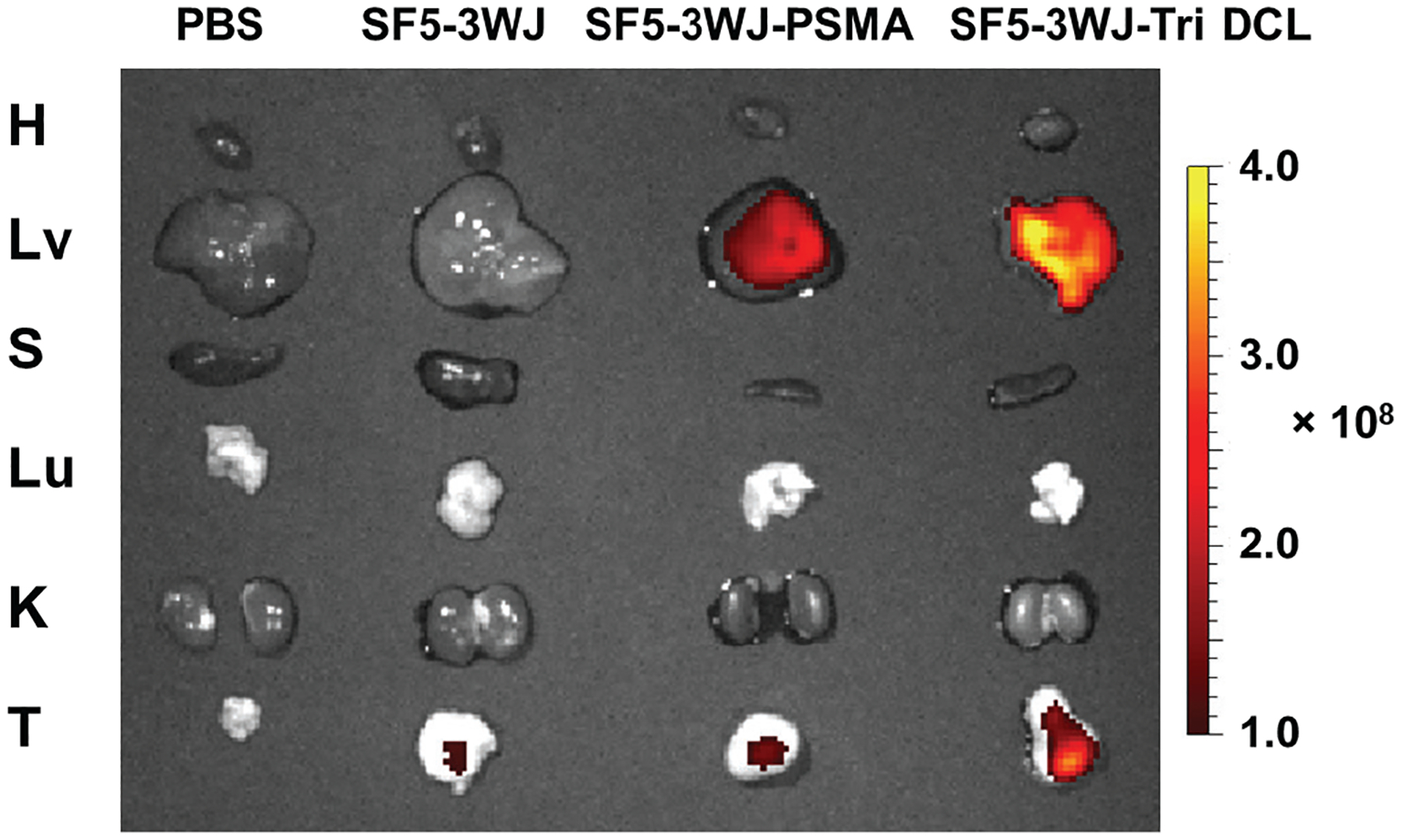
*Ex vivo* fluorescent imaging of organs showing the distribution of SF5 3WJ nanoparticles in PC3 PIP xenograft bearing mice (T: tumor, H: heart, S: spleen, Lu: lung, K: kidney, and Lv: liver).

The inclusion of PSMA targeting ligands should improve the *in vivo* tumor accumulation and targeting^15^. Fluorescently labeled SF5 3WJ RNA nanoparticles (AFdye 647) were intravenously injected into PCa tumor bearing mice via tail-vein injection. At 8 h post-injection, vital organs and tumor were taken out from mice for fluorescent imaging (Fig. 6). In the PC3 PIP xenograft-bearing nude mice, SF5 3WJ-Tri DCL showed substantial tumor accumulation compared to SF5 3WJ and SF5 3WJ-PSMA nanoparticles. However, the liver accumulation was also found to be more significant in SF5 3WJ-Tri DCL groups (Fig. 6). Interestingly, the SF5 3WJ-PSMA also exhibited some liver accumulation which may be explained by the increased overall hydrodynamic size of the nanoparticle. These results demonstrate the capability of the RNA nanoparticles to specifically target and identify PCa tumors by RNA nanoparticles and could be used in diagnostic applications.

### *In vivo* PET/CT imaging using radiolabeled RNA nanoparticles

Small PSMA-targeted molecules labeled with ^68^Ga and ^18^F have been developed for primary staging and detection of tumors at biochemical recurrence using Positron Emission Tomography/Computed Tomography (PET/CT)^3,4,7^. To test the capabilities of the SF5 3WJ nanoparticles to serve as PET/CT imaging agents, radiolabeled RNA nanoparticles were delivered to mice harboring PCa tumors and time course imaged using a PET/CT scanner.

**Fig. 7.**
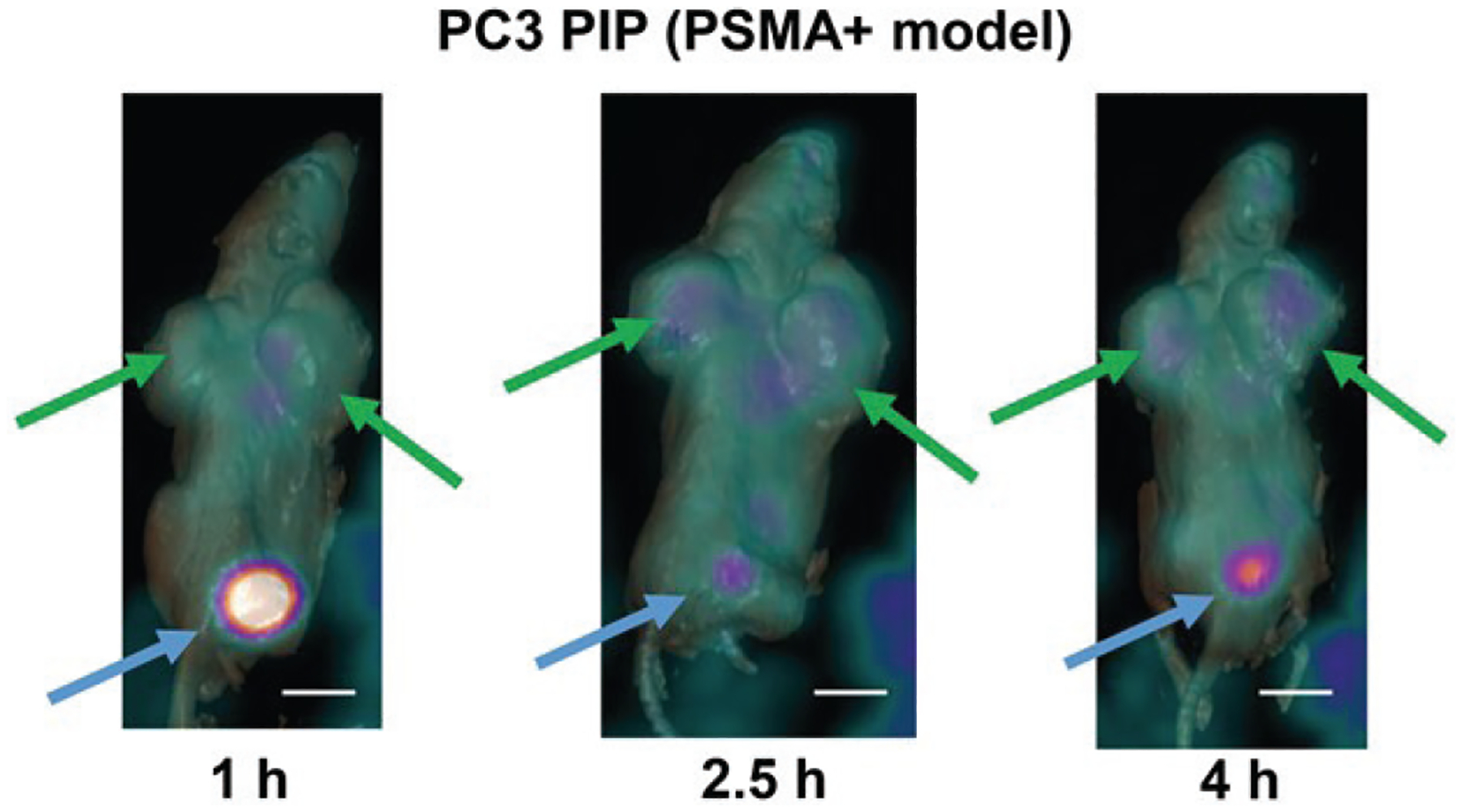
*PET imaging* of prostate cancer in PC3 PIP xenograft bearing mice (Green arrows indicate the location of PC3 xenograft and the blue arrows indicate the bladder). Scale bar – 1 cm.

Male BALB/c nude mice bearing PC3 PIP tumors were injected with [^68^Ga]-SF5 3WJ-NOTA, and were imaged by PET/CT. The radioactivity was highly accumulated in tumor and bladder (Fig. 7). At 1 h post-injection, marked activity was observed in bladder and kidney, indicating the rapid renal clearance of the [^68^Ga]-SF5 3WJ-NOTA. On the images of [^68^Ga]-SF5 3WJ-NOTA at 2.5 h and 4 h, the radiopharmaceutical uptake of the tumor was clearly enhanced, suggesting the increased accumulation in PC3 PIP tumor. Moreover, less liver accumulation was observed at 4 h compared to the 2.5 h time point. The PET/CT imaging results demonstrated the targeting of RNA nanoparticles to PCa *in vivo* and showed potential for diagnosing prostate cancer tumors. Given the spontaneous tumor accumulation of RNA nanoparticles, they can be applied for diagnosis of many tumor types and radiotherapy of various cancers.

### Rapid clearance of RNA nanoparticle imaging marker and radioisotope from the body

A key component to achieving a high tumor to background signal ratio is the rapid clearance of imaging agent from the body to prevent background signal formation. Fig. 1 revealed that the RNA nanoparticles are cleared rapidly from the body, through comparison of the ratio of the fluorescence marker in liver to tumor between 8 and 16 hours. Additionally, [^68^Ga]-SF5 3WJ-NOTA demonstrated signal overlap with the bladder of mice (Fig. 7, 1 h and 4 h) indicating rapid filtration of the RNA nanoparticle through the kidneys. The motile and deformative property of the 3WJ nanoparticles led to rapid clearance of imaging marker and radioisotope from the body.

## DISCUSSION

RNA nanoparticles have previously demonstrated as efficient drug delivery vehicles due to their high stability and dynamic and deformable properties^27,31,37,38,50^. This allows for RNA nanoparticles, including small 3WJs and larger nanoconstructs to spontaneously accumulate in tumors during body circulation by deforming and squeezing through leaky tumor vasculature^27^. These properties further extend to quick body clearance as the RNA nanoparticles are able to excrete to the urine via glomerular filtration. Thus, the deformation of RNA nanoparticles allow for larger (<5 nm) to squeeze through the 5.5 nm size cutoff and clear from the body^12,25,27,28^. It was believed that these biodistribution properties could allow for RNA nanoparticles to serve as an ideal imaging agent and be applied for PET/CT imaging for the diagnosis of various tumors. A key advantage to using this 3WJ nanoparticle platform as an imaging agent is the adaptability of the nanoparticles and multivalency. One strand of the SF5 3WJ can hold the NOTA for radioisotope labeling while a second strand can be interchanged to carry various tumor targeting ligands. Thus, a simple plug-and-play platform is generated to create a library of PET/CT imaging agents by only changing one component strand of the RNA nanoparticle. Therefore, it is not necessary to synthesize an entirely new ligand-NOTA compound like ^68^Ga-PSMA. Additionally, we have shown small RNA nanoparticles like the SF5 3WJ are safe for *in vivo* delivery, as no cytokine induction, immunological responses, or organ toxicities were detected in previous studies^27,28,51^.

Using RNA as a PET/CT imaging agent has proven challenging due to non-specific labeling of radioisotopes to the backbone of RNA. The use of chelating agents such as NOTA and DOTA have become highly popular for generating radioligands to be used in PET imaging. NOTA and DOTA allow for simple and quick inclusion of short-lived radioisotopes following ligand production just prior to delivery to patients. Their use for radiolabeling RNA as an imaging agent has remained a challenge as the phosphodiester backbone of RNA is negatively charged, thus creating charged-charged interactions with the positively charged ^68^Ga. This results in researchers believing the ^68^Ga is chelated to the RNA-NOTA but in fact these labels quickly dissociate from the RNA backbone in *in vivo* conditions. To circumvent these non-specific labeling issues, RNA nanoparticles harboring NOTA were incubated with ^68^Ga at a pH of 3.6 to protonate the negatively charged backbone of RNA. Thus, ^68^Ga was only chelated by NOTA. This chelating process also required an elevated temperature of 90 – 95°C which easily denatures RNA base pairing. The high thermostability and one-pot formation of the SF5 3WJ based RNA nanoparticles (Figure 3C) resulted in complete re-assembly of the nanoparticles as demonstrated by a single peak in the size exclusion chromatography (Fig. 2B). Together the stability of RNA nanoparticles at high temperatures and acidic conditions allowed for ^68^Ga labelling and use as a PET imaging agent.

Here it is demonstrated through fluorescence imaging and PET/CT imaging studies the capability of RNA nanoparticles to spontaneously accumulate in tumors of mice within 1 – 4 hours. Whole body imaging of mice demonstrated an adequate tumor to background signal to positively identify tumors. Early time point imaging shows RNA nanoparticle signal in the kidneys, liver, and lungs but these signals are the RNA nanoparticles circulating in the blood and not actual organ accumulation^42^. Overtime, the lung and liver signals reduce and kidney signal is reduced as the RNA nanoparticles are filtered to the urine and excreted by mice^27,28,42^. These results closely match our previous quantitative biodistribution studies of 3WJ nanoparticles in mice^35^. These studies compared 3WJ nanoparticle accumulation in tumors to healthy organs where it was found 5% of delivered dose accumulated in the tumor with only other accumulation seen in kidney and liver. The highest tumor accumulation was seen around 4 h post injection while signal in healthy organs stabilized after 1 h producing a low background signal^35^. Fluorescent imaging of multiple RNA nanoparticles demonstrated the versatility of the technology and ability of a variety of RNA nanoparticles to be used in cancer imaging. Branched dendrimer structures can be used to include multiple NOTA conjugates to increase radioisotope labeling as demonstrated here with multiple iFluor 647 fluorophores^25^. Additionally, the size and shape of RNA nanoparticles can be tuned as demonstrated by the RNA square from 5 to 20 nm which can be used to tune the circulation time of the nanoparticle to optimize the tumor to background signal^20^. Generally, the results support that a smaller nanoparticle results in faster tumor accumulation and body clearance for clearer imaging, but this may not always be the case.

As shown by our results RNA nanoparticles can harbor tumor targeting ligands. In this case, PSMA expressed on prostate cancer cells was effectively targeted by an RNA aptamer or chemical ligand on the RNA nanoparticle. Inclusion of such ligands allow for enhanced tumor targeting and allow for improved specific tumor diagnosis as shown in Fig. 6 comparing SF5 3WJ signal to SF5 3WJ-Tri DLC. However, during patient examination for early cancer diagnosis, a specific tumor type or target receptor may not be known. The accumulation of RNA nanoparticles without tumor targeting ligands as shown in Figs. 1 & 7 demonstrates the versatility of RNA nanoparticles to identify multiple tumor types.

These results demonstrate a proof of concept of RNA nanotechnology serving as a possible PET imaging agent for noninvasive early tumor detection. Further testing of RNA nanoparticle PET agents will need to be conducted to optimize RNA nanoparticle used, isotope dose, and inclusion of tumor targeting ligands to advance this technology to the clinic. is an FDA approved imaging agent that targets PSMA such as Piflufolastat F-18 (18F-DCFPyL), Fluorine-18 flotufolasta, and Gallium-68 gozetotide have shown dramatic improvement in PET imaging for detection of prostate tumors^46,52–56^. While the results here are promising, it is likely that the radiolabeled SF5 3WJ nanoparticles do not perform as well as 18F-DCFPyL and as this proof-of-concept needs further refinement and optimization to match such a well-developed imaging agent.

## CONCLUSIONS

Here, the construction of SF5 3WJ-based RNA nanoparticles as novel PET/CT imaging agents is reported. These RNA nanoparticles demonstrate high ^68^Ga labeling and specifically only on NOTA labels. SF5 3WJ nanoparticles demonstrated high targeting to prostate cancer cells and accumulation into prostate cancer tumors expressing PSMA through controllable valency of DCL ligands. *In vivo* fluorescent imaging in KB tumors and PC3 PIP prostate tumors in mice demonstrated spontaneous tumor targeting while having very low to no organ accumulation. By radiolabeling of RNA nanoparticles, *in vivo* PET/CT imaging of prostate tumors was achieved within the first 4 hours of RNA nanoparticle delivery, indicating the accumulation of RNA nanoparticles in tumor. Results demonstrate the feasibility and promise of RNA nanoparticles to serve as PET/CT imaging agents. The proof-of-concept study is an important step in the direction of developing novel radiotherapeutic agents for various cancers by conjugating the RNA nanoparticle with a suitable chelating agent and radiolabeling with a radionuclide, the list includes ^177^Lu, ^225^Ac, ^212^Pb, ^213^Bi, ^223^Ra, and ^90^Y.

## MATERIALS AND METHODS

4-Dimethylaminopyridine (DMAP), Copper(I) Bromide (CuBr), Tris[(1-benzyl-1H-1,2,3-triazol-4-yl) methyl]amine (TBTA) were purchased from SigmaAldrich (St. Louis, MO). 1-Ethyl-3-(3-dimethylaminopropyl) carbodiimide hydrochloride (EDC) was obtained from Thermo Fisher Scientific (Rockford, IL). 6-Azidohexonic acid was ordered from Chem-Impex International, Inc (Wood Dale, IL). All solvents for organic synthesis were supplied by Sigma-Aldrich (St. Louis, MO). Chemicals, solvents and other supplies for solid-phase oligonucleotide synthesis and HPLC were purchased from Bioautomation (Irving, TX) and Glen Research (Sterling, VA). Chemicals for gel electrophoresis were purchased from Fisher Scientific (Fair Lawn, NJ) and Bio-Rad (Hercules, CA). Mediums and buffers for cell culture were supplied by Thermo Scientific and Sigma-Aldrich. 4–5 weeks male athymic nu/nu mice were purchased from Taconic (Rensselaer, NY).

### Synthesis of DCL-azido

A solution of DCL (8 mg) in DMSO (250 μL) was added to *N,N*-diisopropylethylamine (0.08 mL, 0.47 mmol), followed by NHS-azido (7 mg). After incubation at room temperature for 4 h, the reaction mixture was directly used for conjugation with alkyne-modified RNA.

### Synthesis of RNA oligos

Solid-phase RNA oligomer synthesis was performed on a 1 μmole scale. An automated oligo synthesizer ASM-800E from Biosset was used to start the synthesis from the universal 1,000 Å long chain amino alkyl-controlled pore glass (LCAA-CPG) solid support. Coupling efficiency was monitored after removal of the dimethoxytrityl (DMT) protecting groups. Each strand was synthesized using 2′-fluorinated (2′-F) pyrimidines. Following synthesis, oligomers were cleaved from beads and deprotected in a 1:1 mixture (v/v) of ammonium hydroxide and methylamine (AMA) solution at RT for 2 h. The 2′-TBDMS protecting groups were removed by triethylamine trihydrofluoride (TEA.3HF) followed by desalting using Glen gel-pak desalting column. The collected fraction was dried under speed vacuum. Sequences of all RNA oligos synthesized can be found below. Sequences of the RNA oligomers used are listed in 5′ to 3′ orientation. The synthesized strands were analyzed using 8 M urea 16% denaturing PAGE.

**Table:** 

SF5 3WJ_A_	- CCUAUUCAGGUGCGUGCUGGUGCUACCGAUGUAAUUCAA
SF5 3WJ_A_-PSMA	- AAGGGACCGAAAAAGACCUGACUUCUAUACUAAGUCUACGUUCCCUUUUUUCCUAUUCAGGUGCGUGCUGGUGCUACCGAUGUAAUUCAA
SF5 3WJ_A_-Tri-Alkyne	- CCUAUUCAGGUGCGUGCUGGUGCUACCGAUGUAAUUCAA
SF5–3WJ_A_ Bi-Alkyne	- CCUAUUCAGGUGCGUGCUGGUGCUACCGAUGUAAUUCAA
SF5 3WJ_B_	- CCUAUUCAGGUGCGUGCUGGUGCUACCGAUGUAAUUCAA
SF5 3WJ_C_	CCUAUUCAGGUGCGUGCUGGUGCUACCGAUGUAAUUCAA
Phi29 3WJ_A_	- CCUAUUCAGGUGCGUGCUGGUGCUACCGAUGUAAUUCAA
Phi29 3WJ_B_	- CCUAUUCAGGUGCGUGCUGGUGCUACCGAUGUAAUUCAA
Phi29 3WJ_C_	- CCUAUUCAGGUGCGUGCUGGUGCUACCGAUGUAAUUCAA
3WJ H_0_-a/WT-b	- CCUAUUCAGGUGCGUGCUGGUGCUACCGAUGUAAUUCAA
3WJ H_0_-b/WT-b	- CCUAUUCAGGUGCGUGCUGGUGCUACCGAUGUAAUUCAA
3WJ H_0_-c/WT-b	- CCUAUUCAGGUGCGUGCUGGUGCUACCGAUGUAAUUCAA

### Conjugation of multivalent DCL on RNA

DCL-azide was conjugated to alkyne-modified RNA oligos *via* Copper-catalyzed azide-alkyne cycloaddition (CuAAC)^17,37^. 100 mM CuBr solution (tBuOH: DMSO=1:3) was mixed with 100 mM TBTA (tBuOH: DMSO=1:3) in a ratio of 1:2 to yield Cu-TBTA complex. 10 μL of such freshly prepared Cu-TBTA solution was added to 60 μL DCL-azide solution (DMSO, 20 mM) followed by adding 50 μL alkyne-modified RNA oligos solution (water, 1–2.5 mM measured by absorbance at 260 nm). The reaction ran overnight at room temperature. Water was added to reach 400 μL (total volume) followed by adding 40 μL 3 M sodium acetate (pH 5.2) and 1100 μL ethanol. The tube was stored at −20°C for at least two hours to precipitate RNA-DCL conjugate. After centrifugation at maximal speed (13,100 RPM) at 4°C for 30 minutes and washing the pellet with 70% cold ethanol, the pellet was dried by speed vacuum. 100 μL water was used to resuspend RNA-DCL conjugate. RNA-DCL conjugate was further purified by reverse phase HPLC (Solvent A: 0.1 M TEAA (triethylamine acetate) in water and solvent B: 75% acetonitrile and 25% water with 0.1 M TEAA).

### NOTA and fluorophore conjugation on RNA

NOTA NHS ester was purchased from CheMatech and AFdye 647 NHS ester was purchased from Click Chemistry Tools. Conjugation reactions were carried out by mixing a 1:10 molar ratio of primary amine labeled 3WJ_C_: NHS ester in 0.1 M sodium bicarbonate buffer, pH = 8.5. The conjugation reactions were incubated at room temperature for 16 hours while protected from light. Following incubation, the reactions were ethanol precipitated and washed twice with cold 75% ethanol to remove the unreacted NOTA or fluorophore, facilitating purification by HPLC.

### Reverse-phase HPLC purification of RNA-AFdye 647 strand

Fluorophore conjugated RNA was purified using ion-pairing reverse phase (IPRP) HPLC. Gradient mobile phase was used to separate dye-modified RNA from unmodified strands. Buffer A was 0.1 M triethylamine acetate (TEAA) (Glen Research) in water and buffer B was 0.1 M TEAA in 75% acetonitrile and 25% water. All purifications were performed on Agilent PLRP-S column (Agilent Cat. #: PL1512–5500) in Agilent 1260 Infinity HPLC system. A flow rate of 1 mL/min was used throughout all HPLC methods and absorbance was monitored at 260 nm (RNA) and 650 nm (Cy5). Fractions were collected and dried under vacuum and then resuspended in DEPC water. 8M urea 16% denaturing PAGE was used to characterize the purified strands.

### Size-exclusion chromatography HPLC purification of NOTA-conjugated RNA strand

NOTA conjugated RNA was purified from unreacted NOTA using size-exclusion HPLC. HPLC grade water was used as the mobile phase. All purifications were performed on Agilent Bio-SEC column in Agilent 1260 Infinity HPLC system. A flow rate of 0.3 mL/min was used and absorbance was monitored at 260 nm (RNA) and 305 nm (NOTA). Fractions were collected and dried under vacuum and resuspended in DEPC water.

### RNA nanoparticle assembly and purification

Equimolar ratio of composite strands of each RNA nanoparticle were mixed in 1× TS buffer (50 mM Tris base and 100 mM NaCl): 1) SF5 3WJ (SF5 3WJ_A_, 3WJ_B_ and 3WJ_C_)^18^; 2) G3 RNA dendrimer (Phi29 3WJ_A_, Phi29 3WJ_C_, 3WJ H_0_-a/WT-b, 3WJ H_0_-b/WT-b, and 3WJ H_0_-c/WT-b)^25^. By denaturing the strands at 95°C, the mixture solution was slowly cooled down to 4°C at the rate of −2°C/min. The assembled nanoparticles were desalted by Sephadex G50 column to remove salt and small molecule contaminants.

### Radiolabeling of RNA nanoparticles and chelation efficacy determination

1.0 mL of 1.4 mCi ^68^GaCl_3_ (in 0.1 M HCl) was taken in a glass reaction vial and pH adjusted to ~ 3.6 using 1 M NaOAc buffer. 200 μg of the RNA nanoparticles was added and the reaction mixture was concentrated down to 0.5 mL using a combination of heat (90 – 95°C) and nitrogen flow, raising the pH slowly to ~ 5 – 5.5 during this time using NaOAc buffers. Aliquoted radiolabeled sample was then injected into SEC HPLC to determine the radiolabeling efficacy. Agilent Bio SEC-3 100 Å 4.6 × 300 mm was used with 0.15 M NaCl as mobile phase. A UV detector at 260 nm and a radioisotope detector were used to monitor the RNA and ^68^Ga^3+^, respectively. Free ^68^Ga^3+^ comes off the column at around 9 min while RNA complex was eluted at around 6.44 min.

### TGGE characterization

Preassembled RNA nanoparticles were run in a 10% (w/v) native PAGE in TBE buffer at 100 V for 10 min at room temperature^43^. Temperature gradient gel electrophoresis (TGGE) analysis was then performed in a buffer containing 50 mM TRIS pH = 8.0, 100 mM NaCl, and 0.2 mM MgCl_2_ as previously reported. A gradient temperature (40 – 80°C) was applied perpendicular to the electrical current, and the experiment was run for 1 h at 20 W. The gel was stained and visualized as described above. Quantified values of bands for each nanoparticle were divided by the sum of the total values in corresponding lanes, calculated by ImageJ. Melting curves were plotted with quantified data points using GraphPad Prism 8. *T*_*m*_ values were defined as the temperature at which 50% of the loaded nanoparticles dissociated.

### DLS characterization

The apparent hydrodynamic diameter and zeta potential of the RNA nanoparticles in 1 μM in TMS buffer were determined using a Zetasizer Nano-ZS (Malvern Instrument) at 25°C. The laser wavelength was 633 nm. Results were plotted with data points using GraphPad Prism 8 software. Three independent runs with 8 measurements each run were recorded.

### Serum stability characterization of RNA SF5 3WJ-Tri DCL and SF5 3WJ-PSMA samples

Assembled RNA nanoparticles were incubated in cell culture medium containing 10% (v/v) FBS at a final concentration of 1 μM at 37°C. After removal from 37°C incubation after their respective time points, the samples were snap frozen in −80°C to prevent any further degradation. The resulting samples were examined on 6% native PAGE followed by fluorescent imaging by Typhoon imaging system under ethidium bromide and Cy5 channel. Quantification analysis was performed using ImageJ software and plotted by GraphPad Prism 8 software.

### Cell culture

PC3, PC3 PIP, and KB cells were obtained from American Type Culture Collection (ATCC). Each cell line was grown and cultured in RPMI 1640 medium (ThermoFisher Scientific) containing both 10% (v/v) FBS and 1% penicillin/streptomycin at 37°C in humidified air environment containing 5% CO_2_.

### Flow cytometry analysis

100 nM RNA nanoparticles with varying number of DCL harboring AFDye 647 were each incubated with 2 × 10^5^ PC3 and PC3 PIP cells at 37°C for 1 h respectively. After washing with PBS twice, the cells were resuspended in PBS. Flow cytometry analysis was performed by The Ohio State University Analytical Cytometry Shared Resource (ACSR) core facility to compare the binding efficacy of the fluorophore labeled RNA nanoparticles to different cells. The data was analyzed by FlowJo 7.6.1 software.

### Confocal microscopy imaging

PC3 and PC3 PIP cells were seeded on glass cover slips and cultured at 37°C overnight. AFDye 647 labeled RNA nanoparticles were incubated with cells at a final concentration of 100 nM for 1 h at 37°C. The slips were washed twice with PBS buffer followed by fixation using 4% formaldehyde. 0.1% Triton X-100 (Sigma-Aldrich) in PBS buffer was used to treat the slips for 5 min, followed by subsequent cytoskeleton staining using Alexa Fluor 488 phalloidin (ThermoFisher Scientific) for 30 min at room temperature. After rinsing with PBS buffer, the cells were mounted with ProLong@ Gold Antifade Reagent (Life Technologies Corp.) containing DAPI for cell nucleus staining and imaged on Olympus FV3000 confocal microscope (Olympus Corp.). Data were acquired using Fluoview FV31S-SW.

### *In vivo* biodistribution study by fluorescent imaging

RNA nanoparticles were examined for biodistribution profiles to examine time course tumor accumulation and body clearance. All animal procedures were performed in accordance with Subcommittee on Research Animal Care of The Ohio State University guidelines approved by the Institutional Review Board. The protocol for this animal experiment was approved by the Institutional Animal Care and Use Committee (IACUC) of The Ohio State University.

#### Biodistribution of SF5 3WJ nanoparticles to prostate tumors.

80 μL of 15 μM SF5 3WJ RNA nanoparticles fluorescently labeled with AFDye 647 were injected into 5 – 6 week old PC3 PIP tumor bearing male BALB/c mice (Taconic) intravenously (I.V.) through the tail vein. Mice administered with PBS served as blank controls. At 8 h post-injection, mice were sacrificed, and their hearts, kidneys, livers, spleen, and lungs were collected and imaged for Cy5 fluorescent signal using an *In Vivo* Imaging System (IVIS) imager (Caliper Life Sciences).

#### Biodistribution of RNA G3 dendrimers to KB tumors.

KB cell derived tumor xenograft mice model were established by subcutaneously injecting 2 × 10^6^ KB cells in 100 μl PBS into each female athymic nu/nu mice (4 – 6 week old) with folate deficient diet. G_3_ dendrimers conjugated with with iFluor 647 on either the each of the 3WJ H_0_ strands (G_3_-internal) or each of the WT c strands (G3-external) were injected into nude mice bearing KB cell derived xenograft tumor through tail vein injection (100 μl, 20 μM). 1× TS buffer was used as a blank control. Mice were whole body imaged at 1, 4, 8, 16, and 24 hours using Optical imaging system IVIS Lumina II (PerkinElmer Inc.). Mice were sacrificed 8, 16, and 24 h after injection. Major organs, including heart, lungs, liver, spleen and kidneys, together with tumors, were collected and subjected to fluorescence imaging as above.

### *In vivo* PET/CT imaging

To visualize the *in vivo* performance of SF5 3WJ-NOTA/^68^Ga, small animal PET/CT imaging was applied using PC3 PIP xenograft-bearing male nude mice model. Imaging was performed on a Philips Vereos digital PET/CT system. This system is used for clinical imaging, but given the high-resolution image reconstruction, digital PET detection, and fast time-of-flight timing resolution for the system, small animal imaging is readily accomplished. Mice were immobilized on the scanner bed (orientation: prone, nose first) using a 1.5% to 2.5% isoflurane/anaesthetic air mixture for the duration of the scans and were monitored for body temperature and breathing rate. CT X-ray images were acquired for anatomical reference and to allow for scatter and attenuation correction during PET data reconstruction, using the following parameters: 50 KeV, 200 ms, 1:5 binning, and a matrix size of 125 × 125 × 125 μm. PET data were reconstructed with a 1 mm^3^ isotropic voxel size. The *ex vivo* biodistribution study employed administration of 3WJ-NOTA/^68^Ga in mice with the lowest injected dose of 50 μCi per mouse by tail vein injection. PET image data were acquired at 1, 2, 3 and 4 h post-injection.

## Supplementary Material

1
