# Supplementary material for: RNA Nanoparticles Harboring Radioisotopes or Other Imaging Molecules for Spontaneous Tumor Targeting for Early Cancer Diagnosis
Source: RNA Nanomed. Author manuscript; Available in PMC 2025 Oct 16. (PMC12523805; doi:10.59566/isrnn.2025.0201d)
Supplement: 1 [file NIHMS2116912-supplement-1.pdf]

SUPPLEMENTAL FIGURES

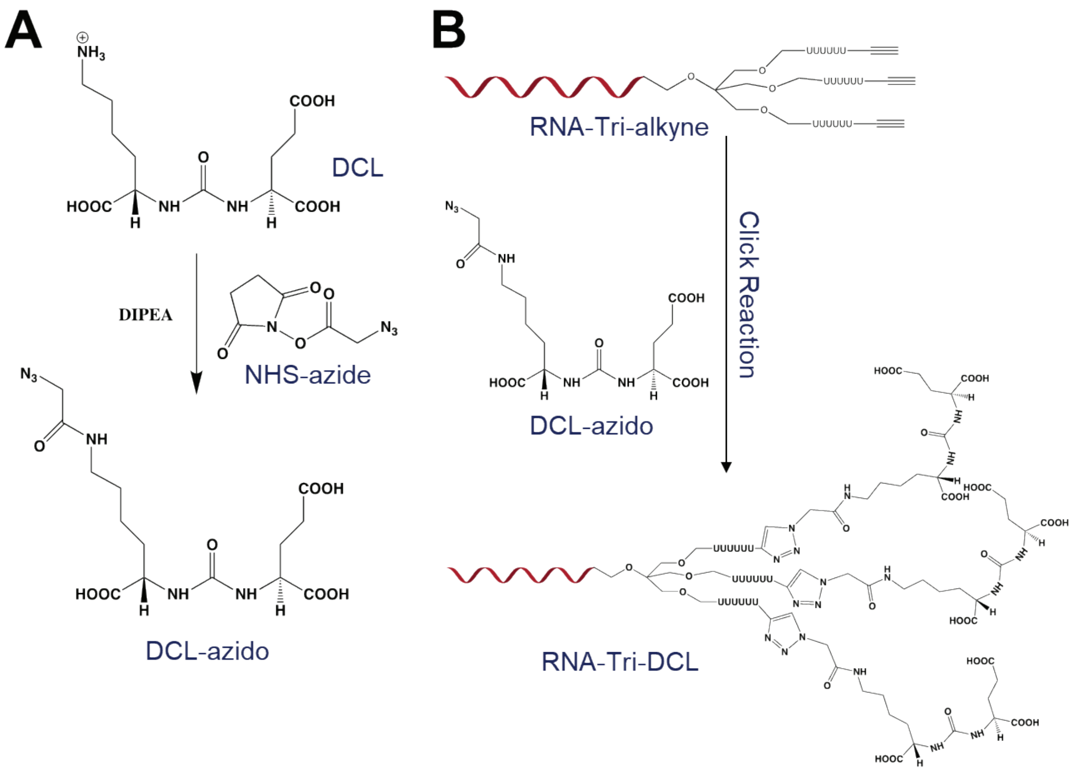

Supplemental Fig. 1. Synthesis Scheme of DCL-N_3 and conjugation of trivalent DCL. (A) Scheme showing the modification of DCL with azido group. **(B)** Reaction scheme showing the modification of trivalent DCL on RNA.

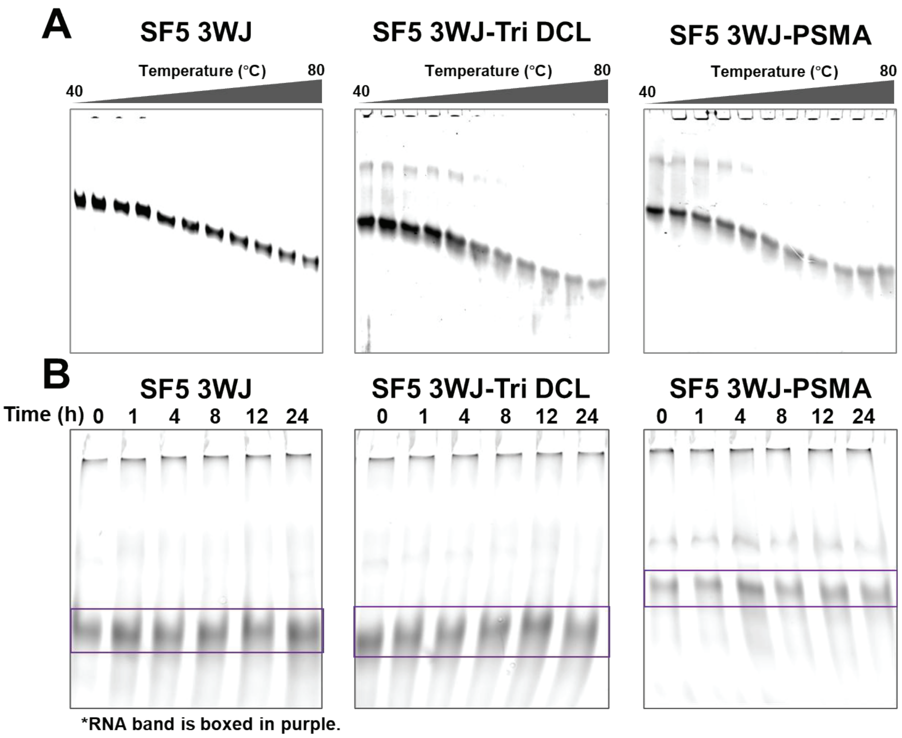

Supplemental Fig. 2. Characterization of PSMA-targeting RNA nanoparticles. (A) Raw 16% Native TGGE gel to quantify SF5 3WJ thermodynamic stability with and without PSMA ligands. **(B)** Representative raw gel 16% Native PAGE of SF5 3WJ nanoparticles following time-course incubation with fetal bovine serum.

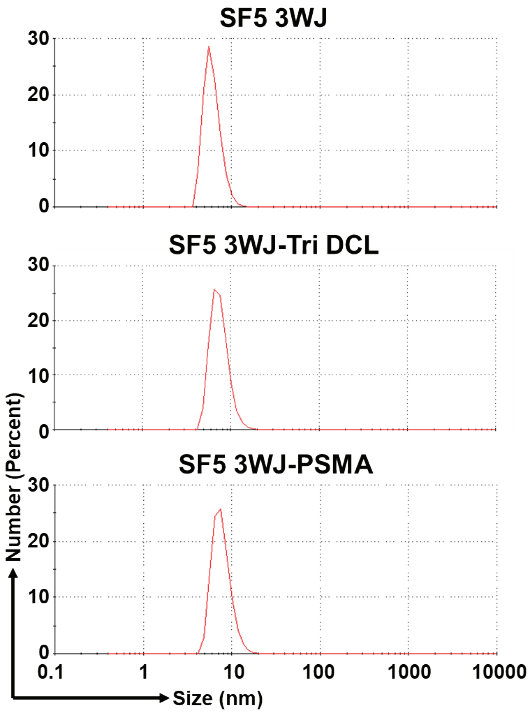

Supplemental Fig. 3. Extended dynamic light scattering results of SF5-3WJs showing no aggregation.
